# Chemoresistance Transmission *via* Exosome-Transferred MMP14 in Pancreatic Cancer

**DOI:** 10.3389/fonc.2022.844648

**Published:** 2022-02-09

**Authors:** Xinyuan Li, Kai Li, Mengmeng Li, Xiaoyu Lin, Yu Mei, Xuemei Huang, Huanjie Yang

**Affiliations:** School of Life Science and Technology, Harbin Institute of Technology, Harbin, China

**Keywords:** MMP14, exosome, gemcitabine resistance, CD44, pancreatic cancer

## Abstract

Pancreatic ductal adenocarcinoma (PDAC) is one of the deadliest malignancies. Gemcitabine is the most commonly used chemotherapy for the treatment of PDAC, but the development of drug resistance still remains challenging. Recently, exosomes have emerged as important mediators for intercellular communication. Exosomes affect recipient cells’ behavior through the engulfed cargos, however the specific cargos responsible for gemcitabine resistance in PDAC are poorly understood. Here, we reported that exosomes could transfer gemcitabine resistance *via* a metalloproteinase 14 (MMP14)-dependent mechanism. MMP14 was identified as a major differentially secreted protein from the gemcitabine-resistant PDAC cells by comparative secretome. It was packaged into the exosomes and transmitted from the chemoresistant cells to the sensitive ones. The exosome-transferred MMP14 could enhance drug resistance and promotes the sphere-formation and migration abilities of the recipient sensitive PDAC cells. Mechanically, exosome-transferred MMP14 promotes the stability of CD44, the cancer stem cell marker in the recipient cells. Our results indicate that MMP14 is a key player for exosome-mediated transfer of gemcitabine resistance, thus targeting MMP14 in exosomes may represent a novel strategy to limit gemcitabine resistance in PDAC.

## Introduction

Pancreatic ductal adenocarcinoma (PDAC) is a devastating human malignancy with an average 5-year survival rate less than 8% (1, 2). Due to early metastasis, most PDAC patients are diagnosed with advanced disease, which are not suitable for surgical resection (3). Gemcitabine represents the first-line treatment of PDAC, but drug resistance is a major obstacle in improving the patient’s response (4).

Recently, exosomes have emerged as important mediators for cell-to-cell communication (5). Exosomes engulf biologically active cargos including proteins, RNAs and lipids, which can be uptaken by adjacent cells and affect their behavior (6). For example, tumor-derived exosome-transferred lncARSR has been reported to transmit sunitinib resistance from drug resistant cancer cells to sensitive ones (7). Exosome-mediated EphA2 transmission transfers gemcitabine resistance in PDAC (8). In addition, exosomes shed from tumor microenvironment were found to promote the stemness, epithelial-mesenchymal transition (EMT), metastasis and chemotherapy resistance of cancer cells (9, 10).

Matrix metalloproteinase 14 (MMP14), also known as membrane-type 1 MMP (MT1-MMP), is a transmembrane Zn^2+^-dependent MMP. MMP14 is localized in the leading edge of migrating cancer cells where it proceeds extracellular matrix (ECM) remodeling by degrading protein components of the ECM and promotes cancer cell migration, invasion and metastasis (11, 12). Studies also indicate that MMP14 regulates cell motility, cancer stemness and other important biological processes non-proteolytically (13–17).

To investigate the role of extracellular components in modulating gemcitabine resistance, we performed LC/MS using parental BxPC-3 and its subline BxPC-3-Gem which developed gemcitabine resistance (18, 19) and found that MMP14 was a major differential protein shed by resistant BxPC-3-Gem cells *versa* parental BxPC-3 cells. As MMP14 is a membrane-bound protein, we hypothesized that MMP14 might be transferred *via* exosomes from chemoresistant PDAC cells, which might affect the chemoresistance of the surrounding PDAC sensitive cells. Thus, we analyzed the influence of exosome-transferred MMP14 on recipient cells’ response to gemcitabine. Our results indicate that MMP14 is a key player for exosome-mediated transfer of gemcitabine resistance.

## Materials and Methods

### Cell Lines and Cell Culture

Human PDAC cell lines BxPC-3 and previously established gemcitabine-resistant subline BxPC-3-Gem (18) were cultured in RPMI-1640 medium (Gibco, BRL Co. Ltd., USA) containing 10% fetal bovine serum (FBS) (Gibco, BRL Co. Ltd., USA)). Mia-PaCa2, PANC-1 and HEK293T cells were cultured in Dulbecco’s Modified Eagle’s Medium (DMEM) (Gibco, BRL Co. Ltd., USA) supplemented with 10% FBS. Cells were cultured in a humid atmosphere containing 5% CO_2_ at 37°C. All cells were tested for mycoplasma at regular intervals.

### Stable Cell Lines

The plasmid pLVSIN-CMV-puro carrying GFP (pLVSIN-GFP) was constructed as described previously (19). pLVSIN-CMV-puro carrying RFP (pLVSIN-RFP) was constructed using the same approach. Stable cell lines were established through lentiviral transduction. Briefly, the constructed vectors and lentiviral packaging mix (VSV-G plasmid and Gag-Pol plasmid) were co-transfected into HEK293T cells. The supernatants containing lentiviruses were collected, filtered, and added into BxPC-3 and BxPC-3-Gem cells for 2 days. The transduced cells (pLVSIN-RFP-BxPC-3-Gem, pLVSIN-GFP-BxPC-3) were selected with puromycin (Santa Cruz, Texas, USA).

### Exosome Purification, Characterization, and Analysis

Exosomes were isolated from PDAC cell lines by ultracentrifugation or PEG. After 48-hour cell culture in exosome-free medium, which was centrifuged at 100 000 g for 70 minutes to remove any exosomes from the serum in advance, the medium was centrifuged at 500 g for 5 minutes to remove any cell contamination. The resulting supernatants were centrifuged at 12 000 g for 20 minutes to remove any possible apoptotic bodies and large cell debris. The exosomes were collected as pellets after ultracentrifugation at 100 000 g for 70 minutes, then washed in PBS and pelleted again by ultracentrifugation.

For PEG approach, appropriate amount of 5X PEG8000 was thoroughly mixed with the 48-hour cell culture medium to a final 1x PEG8000 concentration. After incubation at 4°C for 12 hours, the samples were centrifuged at 12 000 g for 10 minutes. The supernatant was discarded as much as possible and exosome pellets were collected, and then washed in PBS and pelleted again by centrifugation.

Exosome preparations were verified by electron microscopy (Quanta GEG250, FEI, Hillsboro, USA) and the size and particle concentration were analyzed using the ZetaPlus nanoparticle characterization system (Brookhaven, NY, USA).

### Exosome Staining and Quantification

The isolated exosomes were stained with fluorescent dye PKH67 (Sigma, Saint Louis, USA) according to the manufacturer’s protocol. Briefly, exosomal protein concentration was determined by using Pierce BCA Protein Detection Kit (Thermo, Rockford, USA). Exosomes (20 μg) were suspended in 1 mL a Diluent C and incubated with equal volume of Diluent C containing 5 μL of PK67 for 5 min. Serum (2 mL) was added to terminate the staining. After washing with 1×PBS and centrifugation at 4°C, 120,000 g for 70 min, the stained exosomes were re-suspended in 1×PBS.

### Constructs and Transfection

MMP14 overexpression vector (GFP-MMP14) was constructed by cloning the full-length cDNA of MMP14 into the Bgl II/EcoR I sites of pEGFP-C1 vector. The nucleotides targeting MMP14 and negative control were synthesized by GenePharma (Shanghai, China). The sequences of primers and siRNAs are listed in **Table S1**. Transient transfection was mediated by Lipofectamine 3000 (Invitrogen, Eugene, OR, USA) in pLVSIN-RFP-BxPC-3-Gem, pLVSIN-GFP-BxPC-3, PANC-1 and BxPC-3-Gem cells following the manufacturer’s protocol.

### Sphere Formation and Colony Formation Assay

For the sphere formation assay, cells (500/well) were seeded into the ultra-low attachment 6-well plates (Corning, Inc., Corning, NY, USA) and cultured in DMEM-F12 medium (Gibco, Grand Island, NY, USA), containing 2% B27 (Gibco, MD, USA), 10 ng/mL of epidermal growth factor (EGF; Gibco, MD, USA), and 10 ng/mL of basic fibroblast growth factor (FGF; Gibco, MD, USA). After 14 days of culture, the spheres with diameter > 75 μM were counted. For colony formation assay, BxPC-3-GFP and BxPC-3-Gem-RFP cells were seeded into 6-well plates (Corning, Inc., Corning, NY, USA) individually or together, cultured for 14 days, thereafter treated with gemcitabine for 72 hours. The fluorescence of the colonies was detected by the inverted fluorescence microscope (IX71, Olympus Corporation, Japan).

### Transwell Assay

The PDAC cells were seeded in the upper chamber of Transwell (Costar Corp., Cambridge, MA, USA) and allowed to translocate toward medium containing 20% FBS in the lower chamber for 48 hours. 4% formaldehyde and 0.5% crystal violet were used to fix and stain the cells that migrated to the lower surface.

### RT-qPCR and Western Blot

Total RNA was isolated using Trizol (Invitrogen, Eugene, OR, USA) and reverse-transcribed (RT) into cDNA using ReverTra Ace (TOYOBO, Japan). RT-qPCR was performed using SYBR Premix Ex Taq (Takara, Otsu, Shiga, Japan) on ViiA7 Real-time PCR System (Applied Biosystems Inc., Foster City, CA, USA). GAPDH was used as the internal control for mRNA. Detailed information about the primers is shown in **Table S1**.

Cell lysis and Western blot were conducted as previously described (18). Briefly, about 20-40 μg proteins per well were resolved by SDS/PAGE and transferred on PVDF membranes (Millipore, Darmstadt, Germany). The membranes were incubated with antibodies against TSG101 (Proteintech, Wuhan, China), CD44 (Abcam, Cambridge, UK), MMP14 (Abcam, Cambridge, UK), and Activin A (ThermoFisher, Rockford, USA). β-actin (Santa Cruz, CA, USA) was used as loading control. Band intensity was quantified using ImageJ software (NIH).

### Comparative Secretome Analysis

The differentially secreted proteins in BxPC-3-gem *vs* BxPC-3 cells were expressed by fold change, and |log (FC)| > 1 was used as the cut-off value. All statistical analyses were conducted with R software (Version 4.1) and Bioconductor version 4.0. KEGG enrichment and GO function annotation analysis were performed by R package “clusterProfiler” (20). KEGG or GO terms with BH-corrected *p*<0.05 were considered as significance. Gene Set Enrichment Analysis (GSEA) was performed by R package “clusterProfiler” with BH-corrected *p*<0.05 as significance, and “enrichplot” was used to visualize the significant results.

### MTT Assay

The PDAC cells were seeded into 96-well plates and treated with various concentration of gemcitabine (LC Laboratories, Woburn, USA) for 72 hours, followed by the addition of MTT (5 mg/mL, Amresco, USA) for 4 hours. The formed MTT products was dissolved in DMSO (Sigma, Saint Louis, USA), and colorimetric analysis (wavelength, 490 nm) was performed using iMark Microplate Absorbance Reader (Bio-Rad, Hercules, CA, USA).

### Wound Healing Assay

Cells were seeded in 24-well plates (1×10^4^/well) after pre-incubation with exosome. Wound healing assay was described as previously (Wang H., 2018)

### Statistical Analysis

Results for continuous variables are presented as mean ± SD unless stated otherwise. For two-group comparison, we used two-tailed Student’s t test. *p*< 0.05 was considered statistically significant. All analyses were performed using SPSS v.17.0 software (SPSS Inc.).

## Results

### MMP14 Is a Predominant Protein Secreted From Chemoresistant PDAC Cells

**Figure 1:**
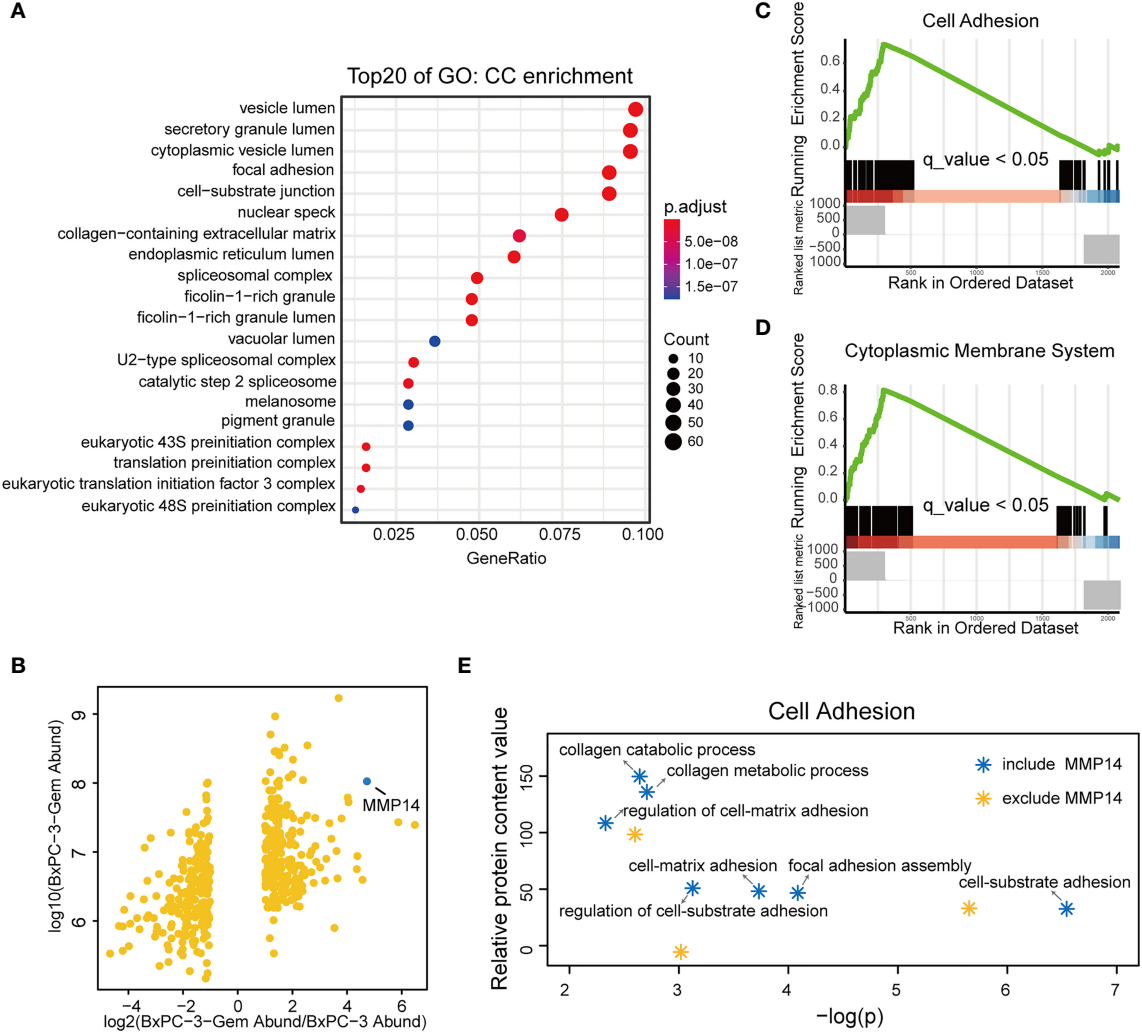
MMP14 was identified as the major differentially secreted protein by comparative secretome. **(A)** Gene Ontology (GO) functional enrichment (Cell Components, CC) for differential proteins in the conditioned medium of BxPC-3-Gem in relative to parental cells. Top 20 functions were exhibited in the plot. **(B)** Scatter plot of differentially secreted proteins in fold change of BxPC-3-GEM vs. BxPC-3 cells (x-axis) against BxPC-3-GEM cells (y-axis). The dot representing MMP14 was shown (blue dot). **(C, D)** GSEA analysis showed that differential proteins in the conditioned medium of BxPC-3-Gem *vs* BxPC-3 cells were enriched in cell adhesion system **(C)** and cytoplasmic membrane system **(D)** (q < 0.05, Bonferroni method; rank ordered by ratio of BxPC-3-Gem/BxPC-3). **(E)** Point plots of molecular functions involved in cell adhesion system. Function pathways included MMP14 (blue stars) or excluded MMP14 (orange star) in cell adhesion system were shown.

Our previous study indicates that the chemoresistant PDAC cells secreted vital factors to enhance the resistance of the sensitive PDAC cells (19). To understand the molecular mechanism, we re-visited and analyzed our comparative secretome data, and obtained the network of proteins based on cell components and molecular function (**Supplementary Figures 1A, B**). More proteins of BxPC-3-Gem cells were associated with cell adhesion, growth factor and receptor ligand activity, cytokine receptor binding, clathrin binding and actin binding (**Supplementary Figures 1A, B**). Gene Ontology (GO) analysis showed that the differential proteins of gemcitabine resistant cells were enriched in vesicle lumen, secretory granule lumen and cytoplasmic vesicle lumen relative to parental cells by cellular component (**Figure 1A**). Because these multivesicular components are involved in the packaging of exosomes (21), this result suggested that some differential proteins in the resistant cells might be secreted *via* exosomes. Among the differential proteins, MMP14 drew our attention because it was among the top 3 differentially expressed proteins in BxPC-3-Gem in relative to parental cells (**Figure 1B**). To compare the functions of the differential proteins, Gene Set Enrichment Analysis (GSEA) analysis was performed. Results showed that BxPC-3-Gem cells secreted more proteins which were associated with cell adhesion and cytoplasmic membrane system relative to BxPC-3 cells (**Figures 1C, D**; **Tables S2****,** **S3**). Analysis of subfunctional pathways involved in cell adhesion system indicated that MMP14 was also related with the regulation of cell-matrix adhesion and other functional pathways (**Figure 1E**; **Supplementary Figure 1C**), indicating that MMP14 is a predominant protein secreted from chemoresistant cells in comparison with the sensitive cells. As MMP14 is a transmembrane protein, it should be engulfed into the exosome for extracellular transmission.

### MMP14 Is Secreted *via* Exosome by PDAC Cells

**Figure 2:**
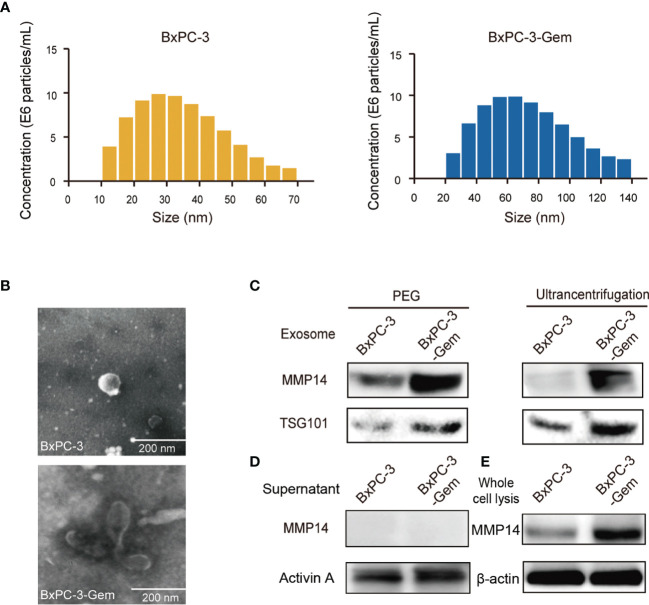
MMP14 was heavily secreted *via* exosome by BxPC-3-Gem cells. **(A)** NanoSight particle tracking analysis of the size distributions and concentration of exosomes extracted from BxPC-3 (left) and BxPC-3-Gem cells (right) by ultracentrifugation. **(B)** Representative electron microscopy images of exosomes secreted by BxPC-3 and BxPC-3-Gem cells by ultracentrifugation. Scale bar, 200 nm. **(C)** Immunoblotting assay of MMP14 expression in the exosomes extracted using PEG and ultracentrifugation methods. TSG101 was used as exosome marker. **(D)** Immunoblotting assay of MMP14 and Activin A expression in the supernatant. Secretory factor Activin A was used as the positive control. **(E)** Immunoblotting assay of MMP14 expression in whole cell lysis. Actin was used as the loading control. Representative images were from three independent experiments.

We isolated all microparticles (microvesicles and exosomes) secreted from BxPC-3-Gem and its parental BxPC-3 cells using differential centrifugation or with polyethylene glycol (PEG) approach. NanoSight particle tracking analysis showed that the predominant microparticles in both BxPC-3-Gem and BxPC-3 cells were of exosomal size (30~100 nm) (6) (**Figure 2A**). The typical size of the exosomes extracted from BxPC-3-Gem ranged 40~110 nm while exosomes isolated from BxPC-3 cells ranged 20~50 nm (**Figure 2A**). In addition, scanning electron microscopy revealed that the isolated microparticles were of exosomal morphology (**Figure 2B**). To see whether MMP14 was engulfed in the exosome, the isolated exosomes were subjected to Western blot. Exosomes were confirmed by the expression of exosome marker, the tumor susceptibility gene (TSG101), while MMP14 was detected in the portion of exosome pellets (**Figure 2C**), but not in the supernatant (**Figure 2D**). Furthermore, more exosomes were secreted from chemoresistant BxPC-3-Gem than parental cells through a quantified analysis of exosomes. Equal amounts of cells were indicated by equal loading control in the two cell lines (**Figures 2C, E**). In addition, higher level of MMP14 was detected in both the exosomes (**Figure 2C**) and whole cell lysates (**Figure 2E**) of the resistant cells in comparison with parental cells.

### Exosome-Transferred MMP14 Confers to Gemcitabine Resistance of Recipient Cells

**Figure 3:**
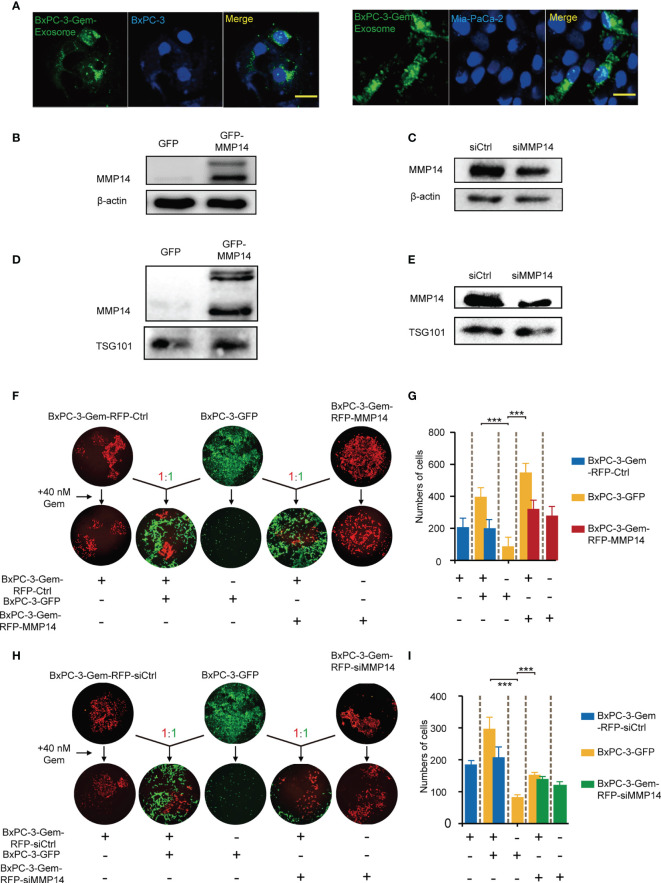
Exosome-transferred MMP14 promotes chemoresistance of the sensitive PDAC cells. **(A)** Fluorescent observation of BxPC-3 and Mia-PaCa-2 cells after 48-hour incubation with PKH67-labeled (green) exosomes derived from BxPC-3-Gem cells, the nucleus of BxPC-3 and Mia-PaCa-2 cells were stained by DAPI (blue). Scale bar, 25 μm. **(B, C)** Immunoblotting assays to test MMP14 overexpression **(B)** or knockdown in BxPC-3-Gem cells **(C)**. **(D, E)** Immunoblotting assays to determine MMP14 levels in the exosomes obtained from the cells described in **(B)**, and **(C) (F, G)** Colony formation in BxPC-3-GFP cells co-cultured with MMP14-overexpressing or control BxPC-3-Gem-RFP cells at a ratio of 1:1 for 2 weeks, followed by treatment with gemcitabine (40 nM) for 72 hours. Representative images from three independent experiments **(F)** and average numbers of colonies **(G)** were shown. **(H, I)** Colony formation of BxPC-3-GFP cells co-cultured with MMP14 knockdown (si-MMP14) or control BxPC-3-Gem-RFP cells at a ratio of 1:1 for 2 weeks, followed by treatment with gemcitabine (40 nM) for 72 hours. Representative images from three independent experiments **(H)** and average numbers of colonies **(I)** were shown. Data in E, G are presented as mean ± SD, ***P < 0.001.

Neighboring cells can uptake exosomes (22). To evaluate whether the exosomes secreted from the resistant PDAC cells can be internalized by the neighboring sensitive cells, BxPC-3 and Mia-PaCa-2cells were incubated with PKH67-dyed exosomes isolated from the gemcitabine-resistant BxPC-3-Gem cells. Using confocal microscopy, we observed PKH67-dyed exosomes internalization into BxPC-3 and Mia-PaCa-2 cells within 48-hour co-incubation (**Figure 3A**). To determine whether exosome-transferred MMP14 could confer the resistant phenotype to recipient sensitive PDAC cells, the exosome donor cells BxPC-3-Gem were overexpressed with MMP14 (**Figure 3B**) or knocked down of MMP14 (**Figure 3C**). The isolated exosomes carried more MMP14 proteins when it was overexpressed, while less exosome-MMP14 proteins were detected when MMP14 was knocked down in BxPC-3-Gem cells compared with the control (**Figures 3D, E**). MTT assay showed that the collected exosomes had no effect on the proliferation of recipient Bx-PC-3 or Mia-PaCa-2 cells upon gemcitabine treatment no matter whether MMP14 was overexpressed or knocked down in the donor resistant cells (**Supplementary Figures 2A, B**). To see whether a long period of incubation with exosomes could educate the recipient sensitive cells in obtaining resistance to gemcitabine, co-culture colony formation assay was performed using GFP-labeled sensitive BxPC-3 cells and RFP-labeled resistant BxPC-3-Gem cells. The BxPC-3 cells became resistant to gemcitabine when co-cultured with gemcitabine-resistant cells for two weeks and MMP14 overexpression in the resistant cells further increased this effect (**Figures 3F, G**). To further address whether exosome-transferred MMP14 was a key intercellular messenger for mediating chemoresistance, MMP14 was knocked down in RFP-labeled BxPC-3-Gem cells. The colony formation of BxPC-3 cells post gemcitabine treatment was increased when co-cultured with RFP-labeled resistant BxPC-3-Gem cells, but was significantly abrogated when MMP14 was knocked down in the resistant cells (**Figures 3H, I**). We noticed that the abrogation effect was comparable to MMP14 knockdown efficiency, indicating that MMP14 is a key player in exosome-mediating transmission of chemoresistance.

### Exosome-Transferred MMP14 Promotes Recipient Cells Sphere-Formation And Migration

**Figure 4:**
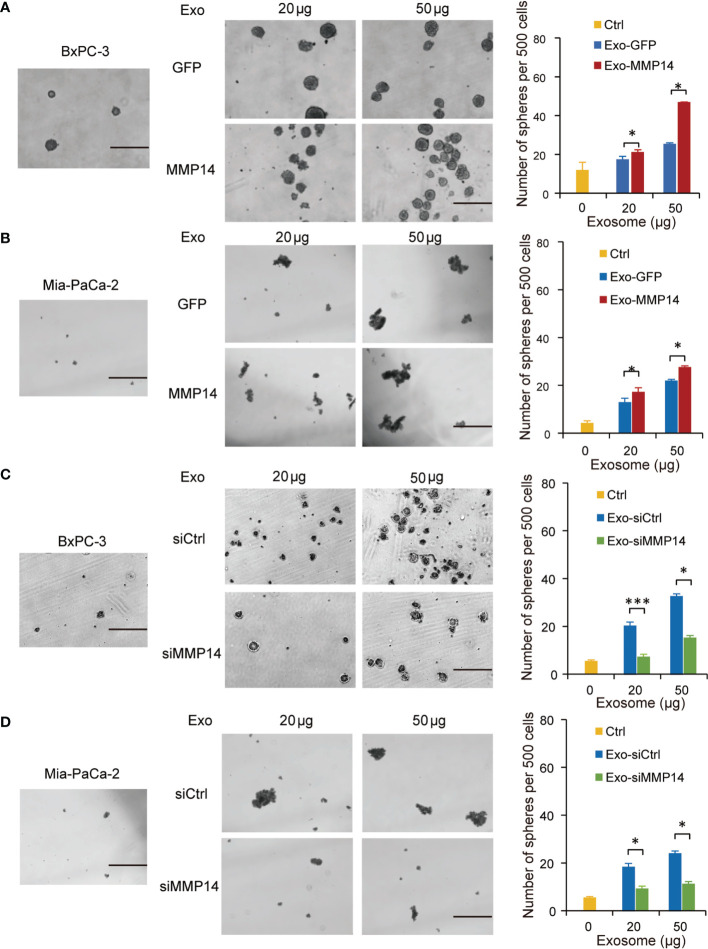
Exosome-transferred MMP14 promotes sphere-formation of the sensitive PDAC cells. **(A, B)** Sphere-forming assay in BxPC-3 **(A)** or Mia-PaCa-2 **(B)** cells incubated with indicated exosomes from MMP14-overexpressing or control PANC-1 cells in a 6-well dish (500 cells per well) for 2 weeks. Representative images (left) from three independent experiments and average number of spheres (right) were shown. **(C, D)** Sphere-forming assay of BxPC-3 **(C)** or Mia-PaCa-2 **(D)** cells with indicated exosomes from MMP14 knockdown or control BxPC-3-Gem cells in a 6-well dish (500 cells per well) for 2 weeks. Representative images (left) from three independent experiments and average number of spheres (right) were shown. Data in A-D are presented as mean ± SD, *P < 0.05, ***P < 0.001. Scale bar, 200 μm.

Cancer cells that acquire cancer stem cell-like properties confer to chemoresistance (23). To determine whether exosome-transferred MMP14 could educate the recipient cells to obtain more aggressive properties, sphere formation assay was performed in the recipient cells post exosome uptake from the resistant cells. The sphere-forming abilities were increased in BxPC-3 and Mia-PaCa2 cells through uptaking exosomes from resistant PANC-1 cells, which were further enhanced when MMP14 was overexpressed in those cells (**Figures 4A, B**). Knockdown approach confirmed that exosome-transferred MMP14 participated in the regulation of cancer stemness of the recipient cells. The sphere-forming abilities were greatly increased in BxPC-3 and Mia-PaCa2 cells by uptaking exosomes from BxPC-3-Gem cells, which were dramatically decreased when MMP14 was knocked down in those cells (**Figures 4C, D**). GSEA enrichment analysis of comparative secretome also showed that MMP14 was involved in the regulation of cell growth and differentiation of stem cells (**Supplementary Figures 3A-C****;** **Tables S4****,** **S5**).

**Figure 5:**
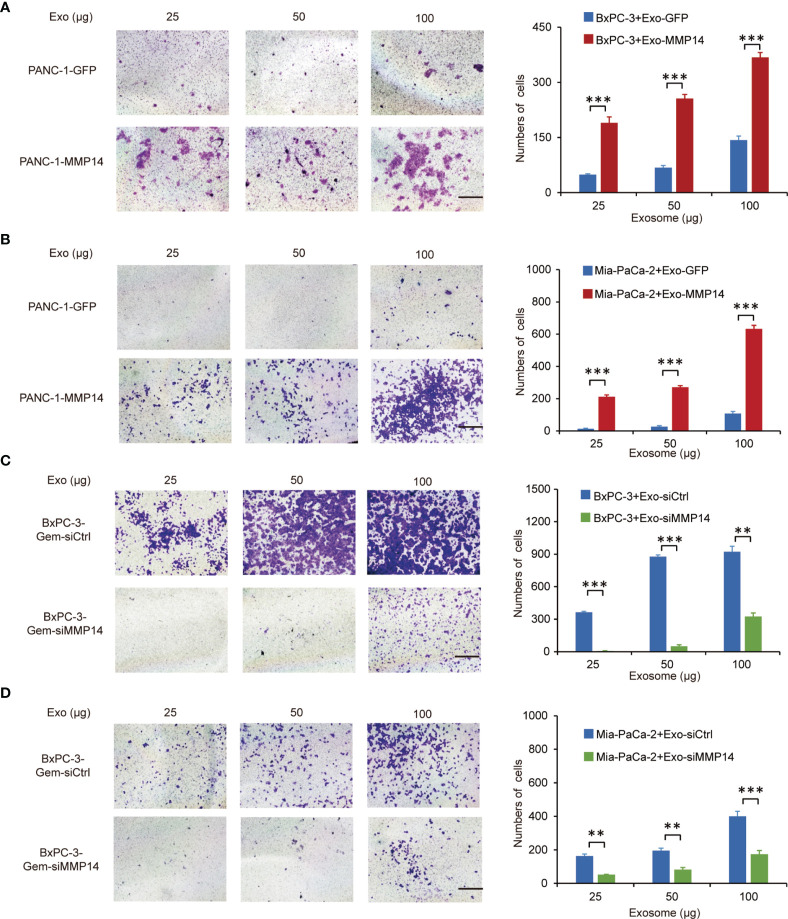
Exosome-transferred MMP14 promotes migration of the sensitive PDAC cells. **(A, B)** Transwell assay in BxPC-3 **(A)** or Mia-PaCa-2 **(B)** cells pre-incubated with indicated exosomes extracted from MMP14-overexpressing or control PANC-1 cells for 48 hours. Representative images from three independent experiments were shown (left) and migrated cells were counted (right). **(C, D)** Transwell assay of BxPC-3 **(C)** or Mia-PaCa-2 **(D)** cells pre-incubated with indicated exosomes extracted from MMP14 knockdown or control BxPC-3-Gem cells for 48 hours. Representative images from three independent experiments were shown (left) and migrated cells were counted (right). Data in A-D are presented as mean ± SD, **P < 0.01, ***P < 0.001.

Transwell assays showed that the exosomes shed by MMP14-overexpressing PANC-1 cells could improve the migration abilities of the sensitive BxPC-3 and Mia-PaCa-2 cells (**Figures 5A, B**). Knockdown approach confirmed that the exosomes from MMP14 knockdown BxPC-3-Gem cells led to decreased migration abilities of BxPC-3 and Mia-PaCa-2 cells in comparison with that from control BxPC-3-Gemcells (**Figures 5C, D**). Scratch assay confirmed that the sensitive cells could obtain increased motility upon receiving exosome-transferred MMP14 (**Supplementary Figures 4A, B**).

### Exosome-Transferred MMP14 Promotes CD44 Stability in the Recipient Cells

**Figure 6:**
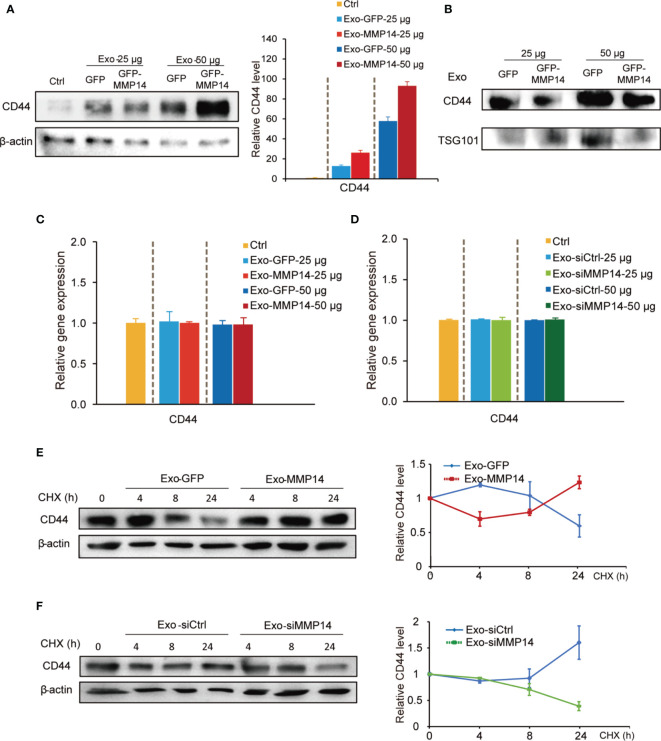
Exosome-transferred MMP14 promotes CD44 stability of the sensitive PDAC cells. **(A)** Immunoblotting assay of CD44 expression in BxPC-3 cells incubated with indicated amounts of exosomes from MMP14-overexpressing or control PANC-1 cells for 48 hours. actin was used as loading control. **(B)** Immunoblotting assay of CD44 expression in the exosomes extracted from MMP14-overexpressing or control PANC-1 cells. The amounts of loading exosomes (25 or 50 µg) were shown. TSG101 was used as the exosome marker. **(C)** RT-qPCR analysis of *CD44* mRNA level in BxPC-3 cells incubated with indicated amounts of exosomes from MMP14-overexpressing or control PANC-1 cells for 48 hours. *CD44* expression in relative to *GAPDH* was normalized to control. **(D)** RT-qPCR analysis of *CD44* mRNA level in BxPC-3 cells incubated with indicated amounts of exosomes from MMP14-knockdown or control BxPC-3-Gem cells. *CD44* expression in relative to *GAPDH* was normalized to control. **(E)** Immunoblotting assay of CD44 expression in BxPC-3 cells incubated with exosomes from MMP14-overexpressing or control PANC-1 cells and treated with CHX (100 µg/mL) for up to 24 hours (left). Quantification of CD44 was normalized to the loading control and expressed relative to 0 hour (right). **(F)** Immunoblotting assay of CD44 expression in BxPC-3 cells incubated with exosomes from MMP14-knockdown or control BxPC-3-Gem cells and treated with CHX (100 µg/mL) for up to 24 hours (left). Quantification of CD44 was normalized to the loading control and expressed relative to 0 hour (right). Representative images above were from three independent experiments. Data in **(E, F)** are presented as mean ± SE.

Epithelial to mesenchymal transition (EMT) is regarded as one of the sources of cancer stem cells (24). Our GSEA analysis and point plots on EMT suggested that both MMP14 and CD44 were involved this process (**Supplementary Figures 4C, D; Table S6**). Given that MMP14 can form a complex with CD44 on the cell membrane (25), we speculated that exosome-transferred MMP14 might affect recipient cells’ stem-like properties *via* modulating the activity of CD44. Western blot showed that the exosomes from either MMP14 overexpressing PANC-1 cells or GFP control cells could increase CD44 protein levels in the recipient BxPC-3 cells (**Figure 6A**). Furthermore, exosomes from MMP14-overexpressing PANC-1 cells led to greater CD44 level in comparison with that from the GFP control cells (**Figure 6A**). We presumed that there are two possibilities associated with CD44 protein increase in the recipient cells. One possibility is the exosomes from MMP14-overexpressing PANC-1 cells might carry more CD44 protein than the GFP control cells. The other possibility is that the exosomes from MMP14-overexpressing PANC-1 cells might lead to more CD44 expression or protein accumulation in the recipient cells than that from GFP control cells. To rule out the possibility that the increased CD44 might have resulted from exosomal transfer, CD44 levels in the exosomes were determined. Results showed that CD44 did exist in the exosomes, however the exosomes from MMP14-overexpressing PANC-1 cells did not carry more CD44 protein in comparison with that from the GFP control cells (**Figure 6B**). mRNA analysis indicated that CD44 mRNA level was not affected by the exosomes (**Figures 6C, D**). In addition, the exosomes from either MMP14-overexpressing PANC-1 or MMP14 knockdown BxPC-3-Gem cells did not change CD44 expression relative to the exosomes from the control cells (**Figures 6C, D**). Then, we used cycloheximide (CHX) to block protein synthesis to see CD44 protein accumulation. The exosomes from MMP14-overexpressing PANC-1 cells led to increase of CD44 protein in the recipient BxPC-3 cells in comparison with the exosomes from GFP control cells (**Figure 6E**). Moreover, the exosomes from MMP14 knockdown BxPC-3-Gem cells led to CD44 protein levels dramatically decreasing in BxPC-3 cells in comparison with the control cells (**Figure 6F**). These results indicate that exosome-transferred MMP14 promotes CD44 stability in recipient cells.

## Discussion

Chemoresistance is the major obstacle for effective interference of PDAC (26). Intercellular communication *via* exosomes represents a key feature of chemoresistance transmission (8). The current study demonstrates that exosome-transferred MMP14 is one of the reasons responsible for gemcitabine resistance in PDAC cells.

MMP14 is a membrane protein (27). Many studies were focused on the proteolytic activity of MMP14 on the cell membrane where it cleaves and activates multiple proteins to promote cancer cell invasion (28, 29). However, few studies were focused on the function of MMP14 in cell-to-cell communication. Research indicated that MMP14 was more enriched in the exosomal fraction of cultured corneal fibroblasts than the cell lysate (30), suggesting that MMP14 is an important molecule in mediating intercellular communication. As a key player, it mediates corneal neovascularization by inducing migration in exosome recipient endothelial cells (31). MMP14 can be shed into extracellular space from cancer cells. Both the full-length 60 kDa and the proteolytically processed 43 kDa forms of MMP14 were detected in the exosomes of fibrosarcoma and melanoma cells (32). In this study, we found that only the full-length MMP14 was engulfed in the exosomes of PDAC cells. The isolated exosomes were identified by their size as well as vesicle structure through electron microscopy and their exosomal marker protein TSG101. In addition, MMP14 was specifically detected in the exosome portion from PDAC cells. Furthermore, PDAC cells resistant to gemcitabine shed more MMP14 *via* exosomes compared with the sensitive parental cells.

Exosomes from cancer cells can be internalized by adjacent cells and affect the recipient cells’ behavior depending on their cargos (33). We found that the uptake of exosome-transferred MMP14 had no influence on the proliferation of the recipient sensitive PDAC cells, however it did educate the sensitive cells in obtaining resistance to gemcitabine after a long period of incubation. Moreover, exosome-transferred MMP14 led to increased cancer stemness and invasion properties in the recipient cells, which are also critical features of chemoresistance (34). Our proteomic analysis indicated that MMP14 was the major protein which was heavily secreted by the resistant cells over the parental sensitive cells. Exosomes from MMP14 knockdown cells lost their abilities to promote stemness and invasion to some extent. Moreover, co-culture with the chemoresistant cells resulted in increased colony formation while knockdown of MMP14 led to dramatic decrease in the sensitive cells post-gemcitabine treatment. These results demonstrate that exosome-transferred MMP14 is a key player for chemoresistance transmission in PDAC. Our results provide new evidence that MMP14 *via* exosome transmission promotes gemcitabine resistance in the sensitive PDAC cells.

CD44 is a common surface marker of cancer stem cells and it plays critical roles in the regulation of stemness and metastasis (35). CD44 functions as a receptor for ECM components such as hyaluronan to activate the Nanog/Stat-3 signaling pathway, granting stem cells with increased abilities of self-renewal and maintenance (36, 37). Several studies revealed the interaction between CD44 and MMP14. For example, CD44 regulates MMP14 expression through Snail, leading to pancreatic cancer cell invasion (38). MMP14 interacts with CD44 by cytoplasmic tails, resulting in CD44 shedding (39). We did not detect CD44 cleavage by the exosome-transferred MMP14 in this study. Instead, we found that exosome-transferred MMP14 increased the protein level of CD44 in the recipient cells. CD44 was detected in the exosomes of PDAC cells, but the exosomes from MMP14-overexpressing cells did not carry more CD44 than the exosomes from control cells. Thus, the possibility that CD44 increased along with MMP14 in exosomal transfer was excluded. Protein stability assay indicated that exosome-transferred MMP14 increased the stability of CD44 protein in the recipient cells. Therefore, our results provide new mechanism that exosome-transferred MMP14 stabilizes CD44 in the recipient cells.

MMP14 is over expressed in various cancers (40, 41). Recent work revealed that MMP14 overexpression *via* knockdown of its repressor potentiated tumor desmoplasia and chemoresistance in colon cancer (42). Collagen-rich fibrosis is also a pronounced feature of PDAC, which confers to the chemodrug resistance (43). These studies suggest the critical role of MMP14 in mediating chemodrug resistance. Our data supports the chemoresistance transmission role of exosome-transferred MMP14 in PDAC, highlighting a promising therapeutic strategy. Targeting MMP14 with antibody demonstrates great efficacy in limiting breast cancer growth and metastasis (44). Future studies are needed to explore whether MMP14 inhibition could overcome gemcitabine resistance in PDAC, thus providing a targeted therapy for PDAC patients. Furthermore, it would be interesting to determine whether the current study could be translated into clinical application by using exosome-transferred MMP14 as a biomarker to predict PDAC patient’s response to gemcitabine.

## Conclusion

In summary, our data demonstrate that the exosome-transferred MMP14 is a key mediator for the transmission of gemcitabine resistance in pancreatic cancer. The targeting of MMP14 in the exosomes may represent a novel strategy to limit gemcitabine resistance in PDAC.

## Data Availability Statement

The original contributions presented in the study are included in the article/**Supplementary Material**. Further inquiries can be directed to the corresponding author.

## Author Contributions

HY conceived the project and wrote the paper. XinL and KL designed and performed the experiments. XinL performed bioinformatics’ analysis. ML and YM helped some Western blot experiments. XH provided technical support. All the authors read and approved the final manuscript.

## Funding

This work was supported by National Natural Science Foundation of China (82172582, 8187249), Natural Science Foundation of Heilongjiang Province (LH2020H072).

## Conflict of Interest

The authors declare that the research was conducted in the absence of any commercial or financial relationships that could be construed as a potential conflict of interest.

## Publisher’s Note

All claims expressed in this article are solely those of the authors and do not necessarily represent those of their affiliated organizations, or those of the publisher, the editors and the reviewers. Any product that may be evaluated in this article, or claim that may be made by its manufacturer, is not guaranteed or endorsed by the publisher.
