# Supplementary material for: Chemoresistance Transmission via Exosome-Transferred MMP14 in Pancreatic Cancer
Source: Front Oncol. 2022 Feb 9;12:844648. doi: 10.3389/fonc.2022.844648 (PMC8865617; doi:10.3389/fonc.2022.844648)
Supplement: Supplementary file 1 [file DataSheet_1.docx]

**Chemoresistance Transmission via Exosome-transferred MMP14 in Pancreatic Cancer**

**Supplementary Figures and legends**


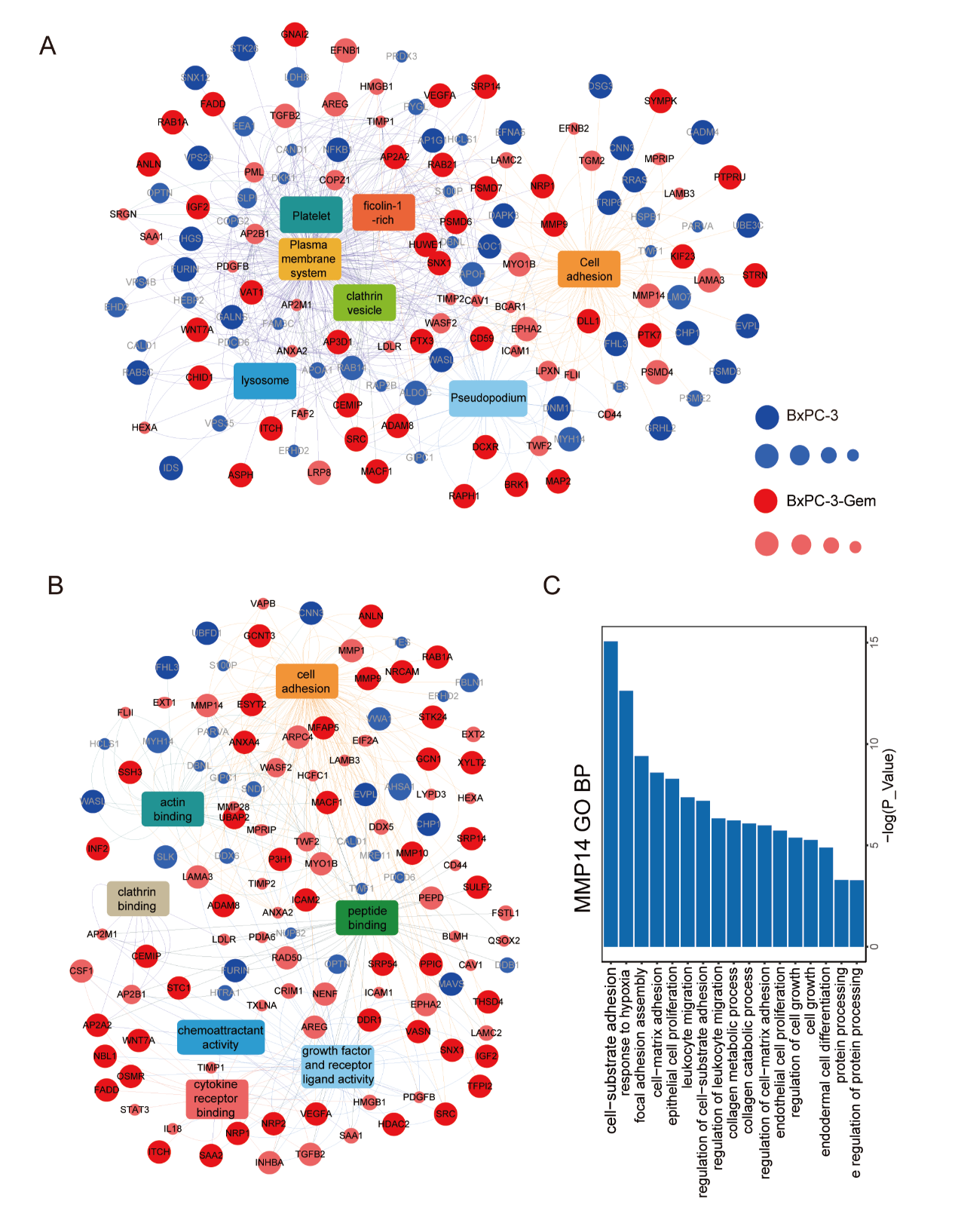


**Supplementary Figure 1.** The network of secreted proteins and GO analysis of MMP14. **(A, B)** The network of proteins based on Cell Components (CC) (A) and Molecular function (B) was obtained from LC-MS/MS data using the conditioned medium of BxPC-3 (blue dots) and BxPC-3-Gem (red dots) cells. R "TCGAbiolinks" was used for analysis. The dot size indicated the content of protein in conditioned medium. **(C)** The distribution bar charts of the GO analysis of biological processes (BP) were shown.


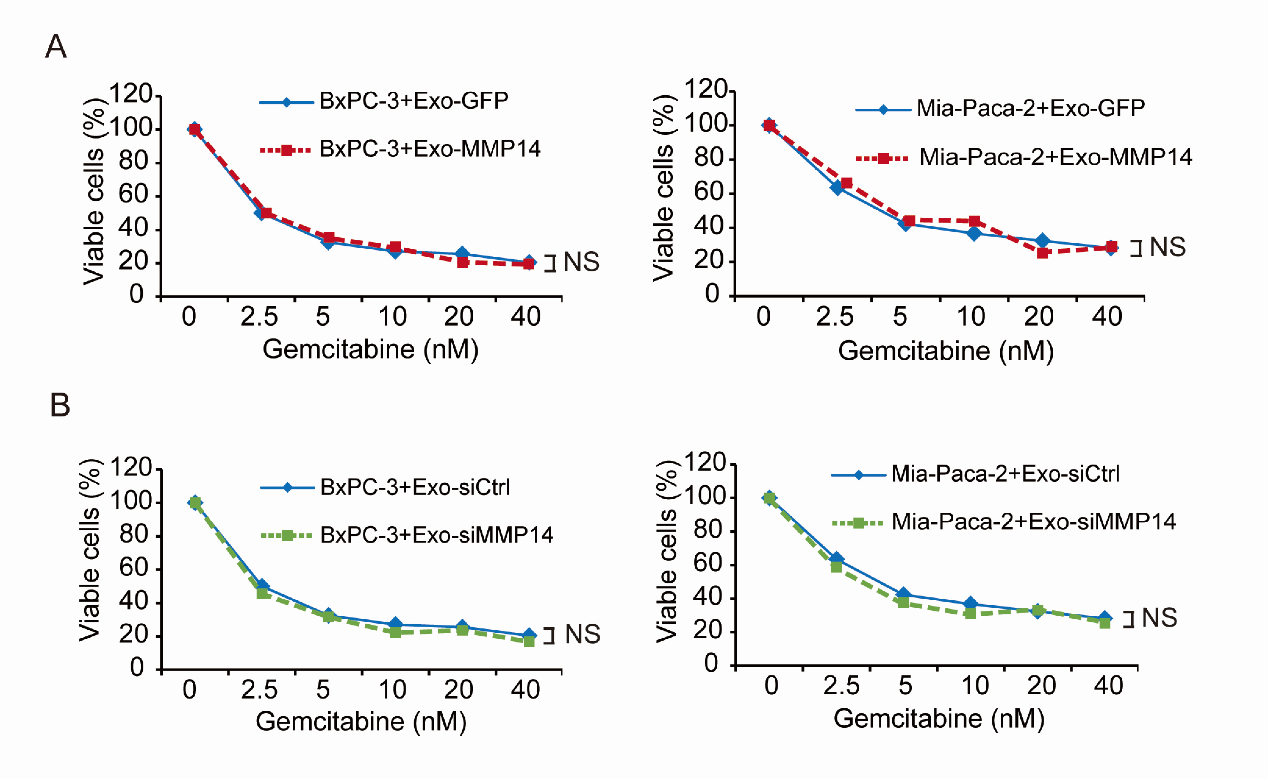


**Supplementary Figure 2.** MTT assays in BxPC-3 and Mia-PaCa-2 cells. **(A, B)** BxPC-3 and Mia-PaCa-2 cells were pre-incubated with indicated exosomes for 48 hours followed by gemcitabine treatment at indicated concentrations for 72 hours (n=3). Exosomes were from PANC-1 cells (MMP14-overexpressing or control) (A) or BxPC-3-Gem cells (siMMP14 or control) (B). Bar, SD. NS., no statistical significance.


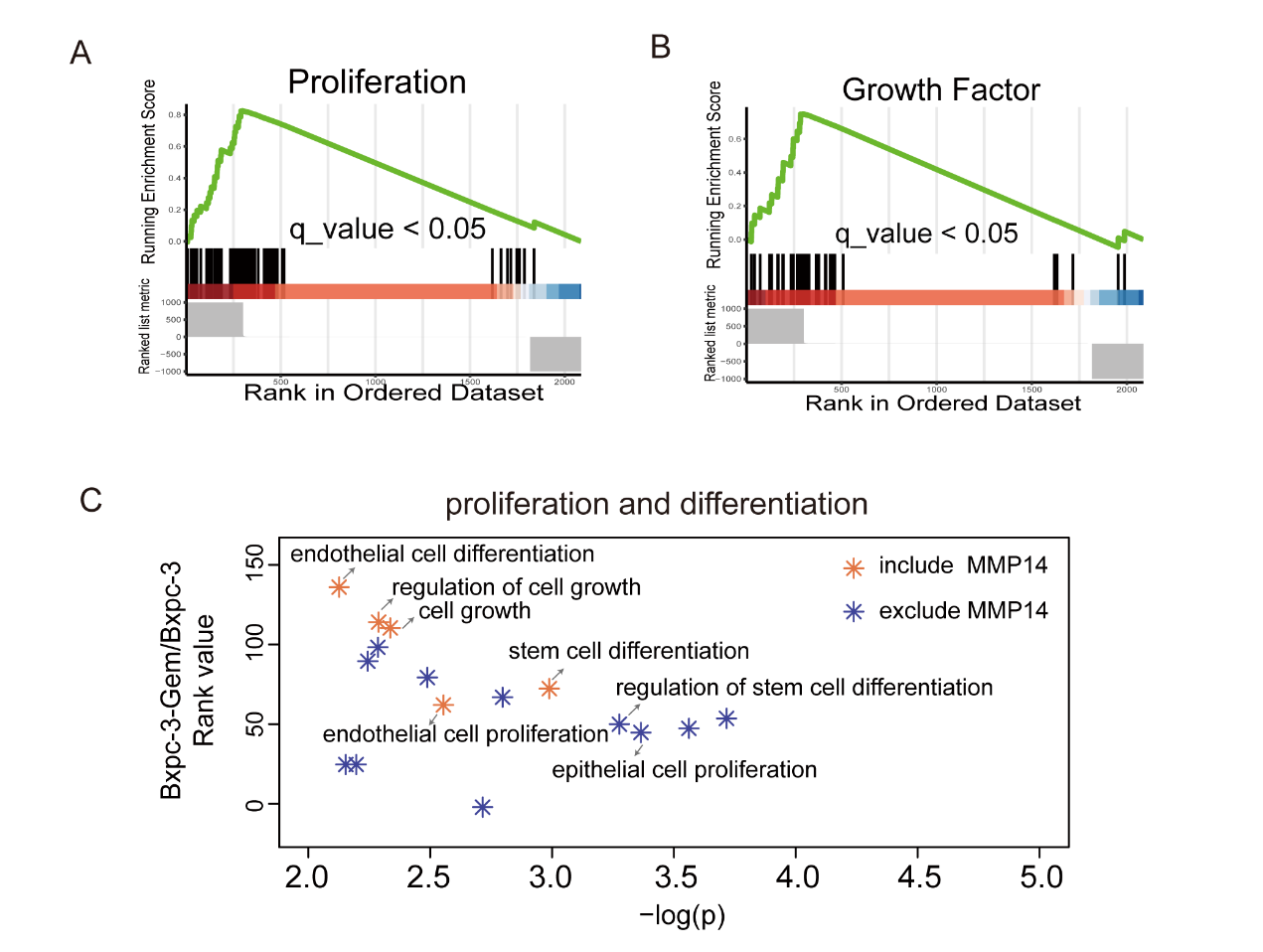


**Supplementary Figure 3.** GSEA analysis. **(A**, **B)** Proteins in the conditioned medium of BxPC-3-Gem were enriched in proliferation pathways (A) and growth factor pathways (B) with q< 0.05 (Bonferroni method; rank ordered by ratio of BxPC-3-Gem/ BxPC-3). (**C)** Point plots of molecular functions involved in proliferation and differentiation.


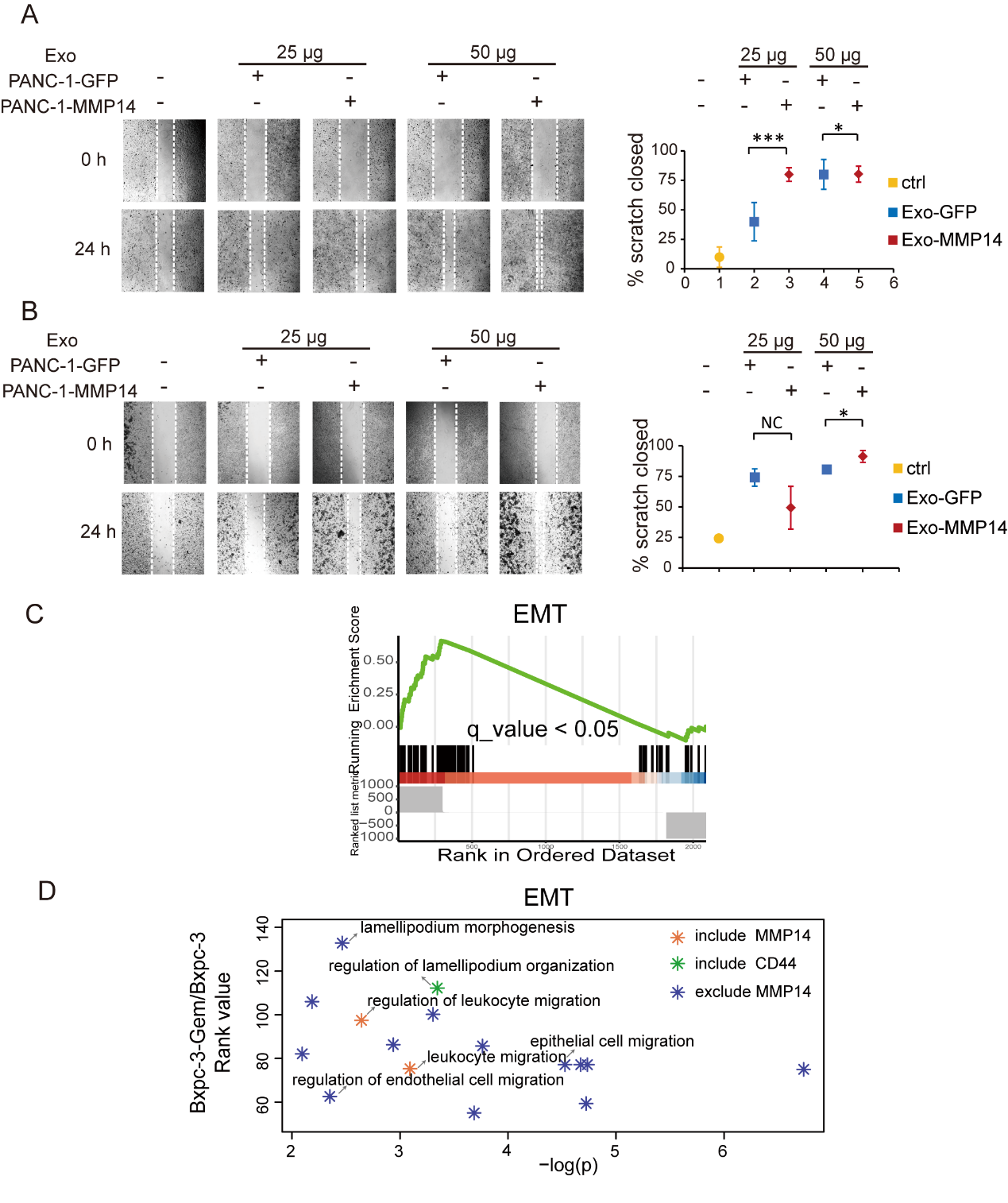


**Supplementary Figure 4.** MMP14 involvement in cell motility. **(A, B)** Wound assay in BxPC-3 (A) and Mia-PaCa-2 (B) cells pre-incubated with indicated exosomes extracted from MMP14 over expression or control PANC-1 cells for 48 hours. Representative images from three independent experiments were shown (left) and migrated cells were counted (right). (**C)** GSEA analysis showed that proteins in the conditioned medium of BxPC-3-Gem were enriched in EMT pathways with q< 0.05 (Bonferroni method; rank ordered by ratio of BxPC-3-Gem/ BxPC-3). **(D)** Point plots of molecular functions involved in EMT regulation. The crucial functions included MMP14 (orange stars) and CD44 (green stars) in EMT processing were shown. Data in A-B are presented as mean ± SD, ***p* < 0.01, ****p* < 0.001.
