# Supplementary material for: Chemoresistance Transmission via Exosome-Transferred MMP14 in Pancreatic Cancer
Source: Front Oncol. 2022 Feb 9;12:844648. doi: 10.3389/fonc.2022.844648 (PMC8865617; doi:10.3389/fonc.2022.844648)
Supplement: Supplementary file 2 [file Table_1.docx]

| Oligonucleotide | Sequence (5’-3’) |
| --- | --- |
| siRNA |  |
| si-NC (negative control) | Sense: UUCUCCGAACGUGUCACGUTT |
|  | Antisense: ACGUGACACGUUCGGAGAATT |
| si-MMP14 | Sense: GCGAUGAAGUCUUCACUUATT |
|  | Antisense: UAAGUGAAGACUUCAUCGCTT |
| RT-qPCR primers |  |
| CD44  GAPDH  Plasmids construction primers  MMP14 | Forward: CTGCCGCTTTGCAGGTGTA  Reverse: CATTGTGGGCAAGGTGCTATT  Forward: TGCACCACCAACTGCTTAGC  Reverse: GGCATGGACTGTGGTCATGAG  Forward: AAAAGATCTATGTCTCCCGCCCCAAGACCCCC  Reverse:  AAAGAATTCTCAGACCTTGTCCAGCAGGGAAC |

**Table S1**. Oligos used for PCR and vector construction
