# Supplementary material for: Chemoresistance Transmission via Exosome-Transferred MMP14 in Pancreatic Cancer
Source: Front Oncol. 2022 Feb 9;12:844648. doi: 10.3389/fonc.2022.844648 (PMC8865617; doi:10.3389/fonc.2022.844648)
Supplement: Supplementary file 3 [file Table_2.docx]

**Table S2.** GSEA analysis of cytoplasmic membrane system(CMS) list in secreted protein components

| Differential protein  (BxPC-3-Gem/ BxPC-3) | | | | | Specific protein  (In BxPC-3 or BxPC-3-Gem) | | | | |
| --- | --- | --- | --- | --- | --- | --- | --- | --- | --- |
| GO | protein | BxPC-3  Abund | BxPC-3  -Gem  Abund | Rank | GO | protein | BxPC-3  Abund | BxPC-3  -Gem  Abund | Rank |
| CMS | MMP14 | 3992373 | 1.06E+08 | 26.48453 | **CMS** | ADAM8 | 0 | 19072996 | 100 |
| CMS | LRP8 | 166423 | 3939355 | 23.67074 | **CMS** | ANLN | 0 | 459959.1 | 100 |
| CMS | AREG | 275272 | 5634323 | 20.4682 | **CMS** | AP2A2 | 0 | 11133384 | 100 |
| CMS | MYO1B | 3783276 | 61542345 | 16.26695 | **CMS** | AP3D1 | 0 | 936237.4 | 100 |
| CMS | TGFB2 | 1703930 | 12200457 | 7.160187 | **CMS** | ASPH | 0 | 3151669 | 100 |
| CMS | LAMA3 | 60007851 | 3.51E+08 | 5.851192 | **CMS** | BRK1 | 0 | 839373.1 | 100 |
| CMS | PSMD4 | 895107 | 4849991 | 5.418337 | **CMS** | CD59 | 0 | 2606039 | 100 |
| CMS | EFNB1 | 21082612 | 1.1E+08 | 5.228169 | **CMS** | CEMIP | 0 | 3054393 | 100 |
| CMS | EPHA2 | 522256 | 2703638 | 5.176844 | **CMS** | DCXR | 0 | 714170.2 | 100 |
| CMS | AP2B1 | 1367589 | 6369102 | 4.657175 | **CMS** | DLL1 | 0 | 2256480 | 100 |
| CMS | COPZ1 | 536602 | 2393501 | 4.460478 | **CMS** | FADD | 0 | 1023999 | 100 |
| CMS | WASF2 | 600335 | 2583093 | 4.302752 | **CMS** | GNAI2 | 0 | 761833 | 100 |
| CMS | TWF2 | 508310 | 2108362 | 4.147789 | **CMS** | HUWE1 | 0 | 1661819 | 100 |
| CMS | LPXN | 7512317 | 30857370 | 4.10757 | **CMS** | IGF2 | 0 | 10946778 | 100 |
| CMS | PML | 1526297 | 6112239 | 4.004619 | **CMS** | ITCH | 0 | 436725.8 | 100 |
| CMS | TGM2 | 33451694 | 1.34E+08 | 4.000107 | **CMS** | KIF23 | 0 | 1537022 | 100 |
| CMS | SAA1 | 3766458 | 14523564 | 3.856027 | **CMS** | MACF1 | 0 | 5646962 | 100 |
| CMS | HMGB1 | 13765277 | 48056378 | 3.49113 | **CMS** | MAP2 | 0 | 717942.8 | 100 |
| CMS | LAMC2 | 99598183 | 3.27E+08 | 3.279849 | **CMS** | MMP9 | 0 | 46062343 | 100 |
| CMS | MPRIP | 12935834 | 41933129 | 3.241625 | **CMS** | NRP1 | 0 | 1031761 | 100 |
| CMS | FLII | 1550861 | 4462020 | 2.877125 | **CMS** | PSMD6 | 0 | 10095984 | 100 |
| CMS | TIMP2 | 31451264 | 89627389 | 2.849723 | **CMS** | PSMD7 | 0 | 2425990 | 100 |
| CMS | LAMB3 | 99246699 | 2.81E+08 | 2.831017 | **CMS** | PTK7 | 0 | 813436.7 | 100 |
| CMS | EFNB2 | 4430595 | 11882958 | 2.682023 | **CMS** | PTPRU | 0 | 1049988 | 100 |
| CMS | LDLR | 10510799 | 27867756 | 2.651345 | **CMS** | PTX3 | 0 | 11448237 | 100 |
| CMS | TIMP1 | 3.59E+08 | 9.27E+08 | 2.582119 | **CMS** | RAB1A | 0 | 2539198 | 100 |
| CMS | AP2M1 | 3107714 | 7921435 | 2.548959 | **CMS** | RAB21 | 0 | 1059247 | 100 |
| CMS | BCAR1 | 932791 | 2375990 | 2.547184 | **CMS** | RAPH1 | 0 | 546824.5 | 100 |
| CMS | CAV1 | 1367988 | 3484054 | 2.546845 | **CMS** | SNX1 | 0 | 1254633 | 100 |
| CMS | ICAM1 | 1629522 | 3912420 | 2.400962 | **CMS** | SRC | 0 | 5656378 | 100 |
| CMS | SRGN | 16343040 | 38616037 | 2.362843 | **CMS** | SRP14 | 0 | 7398565 | 100 |
| CMS | CD44 | 25276155 | 59551622 | 2.35604 | **CMS** | STRN | 0 | 1148684 | 100 |
| CMS | ANXA2 | 2.2E+08 | 5.05E+08 | 2.296067 | **CMS** | SYMPK | 0 | 2402049 | 100 |
| CMS | FAF2 | 740788 | 1621464 | 2.188837 | **CMS** | VAT1 | 0 | 2836647 | 100 |
| CMS | PDGFB | 5245990 | 10736483 | 2.046608 | **CMS** | VEGFA | 0 | 3872900 | 100 |
| CMS | DKK1 | 7675293 | 3741899 | 0.487525 | **CMS** | WNT7A | 0 | 670832.3 | 100 |
| CMS | FAM3C | 2.17E+08 | 1.02E+08 | 0.467989 | **CMS** | CNN3 | 17161001 | 0 | 0 |
| CMS | PARVA | 2768496 | 1269462 | 0.458538 | **CMS** | TRIP6 | 3530861 | 0 | 0 |
| CMS | CALD1 | 10461450 | 4565081 | 0.436372 |  |  |  |  |  |
| CMS | PDCD6 | 11492703 | 4995651 | 0.43468 |  |  |  |  |  |
| CMS | TES | 7690085 | 3231194 | 0.420177 |  |  |  |  |  |
| CMS | GIPC1 | 3260219 | 1336490 | 0.409939 |  |  |  |  |  |
| CMS | PSME2 | 17697264 | 6830125 | 0.385942 |  |  |  |  |  |
| CMS | PYGL | 15746104 | 5493635 | 0.348889 |  |  |  |  |  |
| CMS | LDHB | 62571069 | 18197748 | 0.290833 |  |  |  |  |  |
| CMS | SLPI | 26456208 | 6569537 | 0.248317 |  |  |  |  |  |
| CMS | LMO7 | 7315535 | 1365578 | 0.186668 |  |  |  |  |  |
| CMS | APOH | 4409311 | 811900.7 | 0.184133 |  |  |  |  |  |
| CMS | ALDOC | 1.04E+08 | 19086684 | 0.182896 |  |  |  |  |  |
| CMS | SLK | 6802197 | 368211.9 | 0.054131 |  |  |  |  |  |
