# Supplementary material for: Chemoresistance Transmission via Exosome-Transferred MMP14 in Pancreatic Cancer
Source: Front Oncol. 2022 Feb 9;12:844648. doi: 10.3389/fonc.2022.844648 (PMC8865617; doi:10.3389/fonc.2022.844648)
Supplement: Supplementary file 4 [file Table_3.docx]

**Table S3.** GSEA analysis of cell adhesion (CA) list in secreted protein components

| Differential protein  (BxPC-3-Gem/ BxPC-3) | | | | | Specific protein  (In BxPC-3 or BxPC-3-Gem) | | | | |
| --- | --- | --- | --- | --- | --- | --- | --- | --- | --- |
| GO | protein | BxPC-3  Abund | BxPC-3  -Gem  Abund | Rank | **GO** | protein | BxPC-3  Abund | BxPC-3  -Gem  Abund | Rank |
| CA | INHBA | 276910 | 24694179 | 89.17764 | **CA** | ADAM8 | 0 | 19072996 | 100 |
| CA | MMP14 | 3992373 | 1.06E+08 | 26.48453 | **CA** | ANLN | 0 | 459959.1 | 100 |
| CA | MYO1B | 3783276 | 61542345 | 16.26695 | **CA** | ANXA4 | 0 | 1404352 | 100 |
| CA | MMP1 | 1.32E+08 | 1.71E+09 | 12.91144 | **CA** | AP2A2 | 0 | 11133384 | 100 |
| CA | PEPD | 841981 | 7955964 | 9.449102 | **CA** | CEMIP | 0 | 3054393 | 100 |
| CA | TGFB2 | 1703930 | 12200457 | 7.160187 | **CA** | DDR1 | 0 | 13093354 | 100 |
| CA | ARPC4 | 518481 | 3556722 | 6.859888 | **CA** | ESYT2 | 0 | 2530124 | 100 |
| CA | LAMA3 | 60007851 | 3.51E+08 | 5.851192 | **CA** | GCN1 | 0 | 303997.8 | 100 |
| CA | EPHA2 | 522256 | 2703638 | 5.176844 | **CA** | GCNT3 | 0 | 432138.9 | 100 |
| CA | RAD50 | 633388 | 3247096 | 5.126551 | **CA** | ICAM2 | 0 | 7417062 | 100 |
| CA | AP2B1 | 1367589 | 6369102 | 4.657175 | **CA** | IGF2 | 0 | 10946778 | 100 |
| CA | WASF2 | 600335 | 2583093 | 4.302752 | **CA** | MACF1 | 0 | 5646962 | 100 |
| CA | TWF2 | 508310 | 2108362 | 4.147789 | **CA** | MFAP5 | 0 | 16969022 | 100 |
| CA | SAA1 | 3766458 | 14523564 | 3.856027 | **CA** | MMP10 | 0 | 13474333 | 100 |
| CA | EXT2 | 2059358 | 7759510 | 3.767927 | **CA** | MMP9 | 0 | 46062343 | 100 |
| CA | DDX5 | 3406728 | 12676335 | 3.720971 | **CA** | NRCAM | 0 | 2533520 | 100 |
| CA | FSTL1 | 4761459 | 17409678 | 3.656375 | **CA** | NRP1 | 0 | 1031761 | 100 |
| CA | HMGB1 | 13765277 | 48056378 | 3.49113 | **CA** | NRP2 | 0 | 235674.3 | 100 |
| CA | LAMC2 | 99598183 | 3.27E+08 | 3.279849 | **CA** | P3H1 | 0 | 510053.8 | 100 |
| CA | MPRIP | 12935834 | 41933129 | 3.241625 | **CA** | PPIC | 0 | 408172 | 100 |
| CA | EIF2A | 4208571 | 13072870 | 3.106249 | **CA** | RAB1A | 0 | 2539198 | 100 |
| CA | EXT1 | 1046221 | 3188956 | 3.048072 | **CA** | SNX1 | 0 | 1254633 | 100 |
| CA | MMP28 | 1131350 | 3316110 | 2.931109 | **CA** | SRC | 0 | 5656378 | 100 |
| CA | VAPB | 11409831 | 33095824 | 2.900641 | **CA** | SRP14 | 0 | 7398565 | 100 |
| CA | TIMP2 | 31451264 | 89627389 | 2.849723 | **CA** | SRP54 | 0 | 579419.9 | 100 |
| CA | LAMB3 | 99246699 | 2.81E+08 | 2.831017 | **CA** | STK24 | 0 | 21269953 | 100 |
| CA | PDIA6 | 20534357 | 57807501 | 2.81516 | **CA** | SULF2 | 0 | 1787654 | 100 |
| CA | LDLR | 10510799 | 27867756 | 2.651345 | **CA** | TFPI2 | 0 | 764724.4 | 100 |
| CA | AP2M1 | 3107714 | 7921435 | 2.548959 | **CA** | THSD4 | 0 | 3288651 | 100 |
| CA | CAV1 | 1367988 | 3484054 | 2.546845 | **CA** | UBAP2 | 0 | 979972.1 | 100 |
| CA | QSOX2 | 4698832 | 11717161 | 2.493633 | **CA** | VASN | 0 | 7727365 | 100 |
| CA | ICAM1 | 1629522 | 3912420 | 2.400962 | **CA** | VEGFA | 0 | 3872900 | 100 |
| CA | CD44 | 25276155 | 59551622 | 2.35604 | **CA** | XYLT2 | 0 | 5148303 | 100 |
| CA | LYPD3 | 6040985 | 13998921 | 2.317324 | **CA** | ADAM8 | 0 | 19072996 | 100 |
| CA | ANXA2 | 2.2E+08 | 5.05E+08 | 2.296067 | **CA** | ANLN | 0 | 459959.1 | 100 |
| CA | HEXA | 35087249 | 78264380 | 2.230565 | **CA** | ANXA4 | 0 | 1404352 | 100 |
| CA | HCFC1 | 2471933 | 5079220 | 2.054756 | **CA** | AP2A2 | 0 | 11133384 | 100 |
| CA | PDGFB | 5245990 | 10736483 | 2.046608 | **CA** | CEMIP | 0 | 3054393 | 100 |
| CA | BLMH | 1692049 | 3418083 | 2.020085 | **CA** | CHP1 | 2382711 | 0 | 0 |
| CA | S100P | 2799182 | 1300428 | 0.464574 | **CA** | CNN3 | 17161001 | 0 | 0 |
| CA | PARVA | 2768496 | 1269462 | 0.458538 | **CA** | EVPL | 1145066 | 0 | 0 |
| CA | MRE11 | 3053600 | 1377397 | 0.451073 | **CA** | MAVS | 1784156 | 0 | 0 |
| CA | TWF1 | 8713790 | 3923098 | 0.450217 | **CA** | UBFD1 | 3189437 | 0 | 0 |
| CA | EFHD2 | 10766543 | 4812236 | 0.446962 |  |  |  |  |  |
| CA | CALD1 | 10461450 | 4565081 | 0.436372 |  |  |  |  |  |
| CA | PDCD6 | 11492703 | 4995651 | 0.43468 |  |  |  |  |  |
| CA | NUP62 | 1886161 | 805858.7 | 0.427248 |  |  |  |  |  |
| CA | TES | 7690085 | 3231194 | 0.420177 |  |  |  |  |  |
| CA | GIPC1 | 3260219 | 1336490 | 0.409939 |  |  |  |  |  |
| CA | DBNL | 9172591 | 3753113 | 0.409166 |  |  |  |  |  |
| CA | DDX6 | 3794965 | 1464332 | 0.385862 |  |  |  |  |  |
| CA | SND1 | 29432376 | 10761591 | 0.365638 |  |  |  |  |  |
| CA | DDB1 | 10541455 | 3547273 | 0.336507 |  |  |  |  |  |
| CA | PPIH | 3899545 | 1087905 | 0.278983 |  |  |  |  |  |
| CA | OPTN | 1992779 | 491228.9 | 0.246504 |  |  |  |  |  |
| CA | FBLN1 | 26139569 | 6328828 | 0.242117 |  |  |  |  |  |
| CA | VWA1 | 2991016 | 559307.3 | 0.186996 |  |  |  |  |  |
| CA | APOH | 4409311 | 811900.7 | 0.184133 |  |  |  |  |  |
| CA | AHSA1 | 13897305 | 911345.6 | 0.065577 |  |  |  |  |  |
| CA | SLK | 6802197 | 368211.9 | 0.054131 |  |  |  |  |  |
