# Supplementary material for: Chemoresistance Transmission via Exosome-Transferred MMP14 in Pancreatic Cancer
Source: Front Oncol. 2022 Feb 9;12:844648. doi: 10.3389/fonc.2022.844648 (PMC8865617; doi:10.3389/fonc.2022.844648)
Supplement: Supplementary file 5 [file Table_4.docx]

**Table S4.** GSEA analysis of proliferation and differentiation (P&D) list in secreted protein components

| Differential protein  (BxPC-3-Gem/ BxPC-3) | | | | | Specific protein  (In BxPC-3 or BxPC-3-Gem) | | | | |
| --- | --- | --- | --- | --- | --- | --- | --- | --- | --- |
| GO | protein | BxPC-3  Abund | BxPC-3  -Gem  Abund | Rank | GO | protein | BxPC-3  Abund | BxPC-3  -Gem  Abund | Rank |
| P&D | INHBA | 276910 | 24694179 | 89.17764 | **P&D** | ANLN | 0 | 459959.1 | 100 |
| P&D | MMP14 | 3992373 | 1.06E+08 | 26.48453 | **P&D** | BRK1 | 0 | 839373.1 | 100 |
| P&D | AREG | 275272 | 5634323 | 20.4682 | **P&D** | DDR1 | 0 | 13093354 | 100 |
| P&D | TGFB2 | 1703930 | 12200457 | 7.160187 | **P&D** | DLL1 | 0 | 2256480 | 100 |
| P&D | ARPC4 | 518481 | 3556722 | 6.859888 | **P&D** | GNAI2 | 0 | 761833 | 100 |
| P&D | LAMA3 | 60007851 | 3.51E+08 | 5.851192 | **P&D** | ITCH | 0 | 436725.8 | 100 |
| P&D | PSMD4 | 895107 | 4849991 | 5.418337 | **P&D** | KIF23 | 0 | 1537022 | 100 |
| P&D | EFNB1 | 21082612 | 1.1E+08 | 5.228169 | **P&D** | LOXL2 | 0 | 2103350 | 100 |
| P&D | EPHA2 | 522256 | 2703638 | 5.176844 | **P&D** | MACF1 | 0 | 5646962 | 100 |
| P&D | FSTL3 | 336403 | 1613978 | 4.797751 | **P&D** | MAP2 | 0 | 717942.8 | 100 |
| P&D | WASF2 | 600335 | 2583093 | 4.302752 | **P&D** | MMP9 | 0 | 46062343 | 100 |
| P&D | TWF2 | 508310 | 2108362 | 4.147789 | **P&D** | NRCAM | 0 | 2533520 | 100 |
| P&D | PML | 1526297 | 6112239 | 4.004619 | **P&D** | NRP1 | 0 | 1031761 | 100 |
| P&D | TGM2 | 33451694 | 1.34E+08 | 4.000107 | **P&D** | NRP2 | 0 | 235674.3 | 100 |
| P&D | RBBP7 | 1970032 | 7723151 | 3.920317 | **P&D** | OGFR | 0 | 1020715 | 100 |
| P&D | HMGB1 | 13765277 | 48056378 | 3.49113 | **P&D** | PSMD6 | 0 | 10095984 | 100 |
| P&D | FST | 88042036 | 2.53E+08 | 2.869506 | **P&D** | PSMD7 | 0 | 2425990 | 100 |
| P&D | LAMB3 | 99246699 | 2.81E+08 | 2.831017 | **P&D** | RAB21 | 0 | 1059247 | 100 |
| P&D | IL18 | 9673607 | 27209132 | 2.812718 | **P&D** | RAPH1 | 0 | 546824.5 | 100 |
| P&D | EFNB2 | 4430595 | 11882958 | 2.682023 | **P&D** | SRC | 0 | 5656378 | 100 |
| P&D | BCAR1 | 932791 | 2375990 | 2.547184 | **P&D** | STAG2 | 0 | 1891055 | 100 |
| P&D | CAV1 | 1367988 | 3484054 | 2.546845 | **P&D** | STC1 | 0 | 14519117 | 100 |
| P&D | STAT3 | 923679 | 2274398 | 2.462326 | **P&D** | SULF2 | 0 | 1787654 | 100 |
| P&D | ICAM1 | 1629522 | 3912420 | 2.400962 | **P&D** | TPX2 | 0 | 1597502 | 100 |
| P&D | MYDGF | 2616061 | 6145567 | 2.349168 | **P&D** | VEGFA | 0 | 3872900 | 100 |
| P&D | ANXA2 | 2.2E+08 | 5.05E+08 | 2.296067 | **P&D** | WNT7A | 0 | 670832.3 | 100 |
| P&D | PDGFB | 5245990 | 10736483 | 2.046608 | **P&D** | COMT | 2809818 | 0 | 0 |
| P&D | MCM2 | 3785923 | 7626528 | 2.014444 | **P&D** | DHX36 | 639674 | 0 | 0 |
| P&D | DKK1 | 7675293 | 3741899 | 0.487525 | **P&D** | HMGA2 | 68357 | 0 | 0 |
| P&D | MRE11 | 3053600 | 1377397 | 0.451073 | **P&D** | HSBP1 | 1519808 | 0 | 0 |
| P&D | VPS4B | 2829590 | 1236636 | 0.437037 | **P&D** | KIF2A | 881276 | 0 | 0 |
| P&D | PDCD6 | 11492703 | 4995651 | 0.43468 | **P&D** | KIF4A | 1060392 | 0 | 0 |
| P&D | PSME2 | 17697264 | 6830125 | 0.385942 | **P&D** | PSMD8 | 770871 | 0 | 0 |
| P&D | HTRA1 | 20906638 | 7170785 | 0.342991 | **P&D** | TOP2A | 3016568 | 0 | 0 |
| P&D | PUS7 | 4268973 | 1227475 | 0.287534 |  |  |  |  |  |
| P&D | RFC1 | 4308416 | 1115333 | 0.258873 |  |  |  |  |  |
| P&D | APOH | 4409311 | 811900.7 | 0.184133 |  |  |  |  |  |
