# Supplementary material for: Chemoresistance Transmission via Exosome-Transferred MMP14 in Pancreatic Cancer
Source: Front Oncol. 2022 Feb 9;12:844648. doi: 10.3389/fonc.2022.844648 (PMC8865617; doi:10.3389/fonc.2022.844648)
Supplement: Supplementary file 6 [file Table_5.docx]

**Table S5.** GSEA analysis of growth factor (GF) list in secreted protein components

| Differential protein  (BxPC-3-Gem/ BxPC-3) | | | | | Specific protein  (In BxPC-3 or BxPC-3-Gem) | | | | |
| --- | --- | --- | --- | --- | --- | --- | --- | --- | --- |
| GO | protein | BxPC-3  Abund | BxPC-3  -Gem  Abund | Rank | **GO** | protein | BxPC-3  Abund | BxPC-3  -Gem  Abund | Rank |
| GF | INHBA | 276910 | 24694179 | 89.17764 | **GF** | ANXA4 | 0 | 1404352 | 100 |
| GF | MMP14 | 3992373 | 1.06E+08 | 26.48453 | **GF** | DDR1 | 0 | 13093354 | 100 |
| GF | AREG | 275272 | 5634323 | 20.4682 | **GF** | HDAC2 | 0 | 2452966 | 100 |
| GF | TGFB2 | 1703930 | 12200457 | 7.160187 | **GF** | ICAM2 | 0 | 7417062 | 100 |
| GF | CSF1 | 4368370 | 31146417 | 7.129986 | **GF** | IGF2 | 0 | 10946778 | 100 |
| GF | LAMA3 | 60007851 | 3.51E+08 | 5.851192 | **GF** | NBL1 | 0 | 698604.6 | 100 |
| GF | EPHA2 | 522256 | 2703638 | 5.176844 | **GF** | NRP1 | 0 | 1031761 | 100 |
| GF | NENF | 1811902 | 9091801 | 5.017822 | **GF** | NRP2 | 0 | 235674.3 | 100 |
| GF | CRIM1 | 1487709 | 5746883 | 3.862908 | **GF** | OSMR | 0 | 2142787 | 100 |
| GF | SAA1 | 3766458 | 14523564 | 3.856027 | **GF** | SAA2 | 0 | 596321.1 | 100 |
| GF | HMGB1 | 13765277 | 48056378 | 3.49113 | **GF** | SNX1 | 0 | 1254633 | 100 |
| GF | TIMP2 | 31451264 | 89627389 | 2.849723 | **GF** | SRC | 0 | 5656378 | 100 |
| GF | IL18 | 9673607 | 27209132 | 2.812718 | **GF** | STC1 | 0 | 14519117 | 100 |
| GF | TIMP1 | 3.59E+08 | 9.27E+08 | 2.582119 | **GF** | VASN | 0 | 7727365 | 100 |
| GF | TXLNA | 411516 | 1022661 | 2.485107 | **GF** | VEGFA | 0 | 3872900 | 100 |
| GF | ICAM1 | 1629522 | 3912420 | 2.400962 | **GF** | WNT7A | 0 | 670832.3 | 100 |
| GF | PDGFB | 5245990 | 10736483 | 2.046608 | **GF** | HTRA1 | 20906638 | 7170785 | 0.342991 |
| GF | DKK1 | 7675293 | 3741899 | 0.487525 | **GF** | FURIN | 2795730 | 0 | 0 |
| GF | FAM3C | 2.17E+08 | 1.02E+08 | 0.467989 | **GF** | TRIP6 | 3530861 | 0 | 0 |
| GF | HTRA1 | 20906638 | 7170785 | 0.342991 |  |  |  |  |  |
