# Supplementary material for: Chemoresistance Transmission via Exosome-Transferred MMP14 in Pancreatic Cancer
Source: Front Oncol. 2022 Feb 9;12:844648. doi: 10.3389/fonc.2022.844648 (PMC8865617; doi:10.3389/fonc.2022.844648)
Supplement: Supplementary file 7 [file Table_6.docx]

**Table S6.** GSEA analysis of EMT list in secreted protein components

| Differential protein  (BxPC-3-Gem/ BxPC-3) | | | | | Specific protein  (In BxPC-3 or BxPC-3-Gem) | | | | |
| --- | --- | --- | --- | --- | --- | --- | --- | --- | --- |
| GO | protein | BxPC-3  Abund | BxPC-3  -Gem  Abund | Rank | GO | protein | BxPC-3  Abund | BxPC-3  -Gem  Abund | Rank |
| EMT | MMP14 | 3992373 | 1.06E+08 | 26.48453 | **EMT** | ADAM8 | 0 | 19072996 | 100 |
| EMT | MYO1B | 3783276 | 61542345 | 16.26695 | **EMT** | ANLN | 0 | 459959.1 | 100 |
| EMT | MMP1 | 1.32E+08 | 1.71E+09 | 12.91144 | **EMT** | AP3D1 | 0 | 936237.4 | 100 |
| EMT | PEPD | 841981 | 7955964 | 9.449102 | **EMT** | BRK1 | 0 | 839373.1 | 100 |
| EMT | TGFB2 | 1703930 | 12200457 | 7.160187 | **EMT** | DDR1 | 0 | 13093354 | 100 |
| EMT | CSF1 | 4368370 | 31146417 | 7.129986 | **EMT** | FADD | 0 | 1023999 | 100 |
| EMT | ARPC4 | 518481 | 3556722 | 6.859888 | **EMT** | HDAC2 | 0 | 2452966 | 100 |
| EMT | LAMA3 | 60007851 | 3.51E+08 | 5.851192 | **EMT** | IGF2 | 0 | 10946778 | 100 |
| EMT | EFNB1 | 21082612 | 1.1E+08 | 5.228169 | **EMT** | ITCH | 0 | 436725.8 | 100 |
| EMT | EPHA2 | 522256 | 2703638 | 5.176844 | **EMT** | LOXL2 | 0 | 2103350 | 100 |
| EMT | FSTL3 | 336403 | 1613978 | 4.797751 | **EMT** | MACF1 | 0 | 5646962 | 100 |
| EMT | WASF2 | 600335 | 2583093 | 4.302752 | **EMT** | MMP10 | 0 | 13474333 | 100 |
| EMT | TWF2 | 508310 | 2108362 | 4.147789 | **EMT** | MMP9 | 0 | 46062343 | 100 |
| EMT | PML | 1526297 | 6112239 | 4.004619 | **EMT** | MTA2 | 0 | 9725601 | 100 |
| EMT | SAA1 | 3766458 | 14523564 | 3.856027 | **EMT** | NBL1 | 0 | 698604.6 | 100 |
| EMT | PLOD2 | 2105238 | 7565525 | 3.593667 | **EMT** | NRP1 | 0 | 1031761 | 100 |
| EMT | HMGB1 | 13765277 | 48056378 | 3.49113 | **EMT** | NRP2 | 0 | 235674.3 | 100 |
| EMT | LAMC2 | 99598183 | 3.27E+08 | 3.279849 | **EMT** | P3H1 | 0 | 510053.8 | 100 |
| EMT | MMP28 | 1131350 | 3316110 | 2.931109 | **EMT** | PTPRU | 0 | 1049988 | 100 |
| EMT | LAMB3 | 99246699 | 2.81E+08 | 2.831017 | **EMT** | PTX3 | 0 | 11448237 | 100 |
| EMT | IL18 | 9673607 | 27209132 | 2.812718 | **EMT** | RAB1A | 0 | 2539198 | 100 |
| EMT | EFNB2 | 4430595 | 11882958 | 2.682023 | **EMT** | SAA2 | 0 | 596321.1 | 100 |
| EMT | LDLR | 10510799 | 27867756 | 2.651345 | **EMT** | SNX1 | 0 | 1254633 | 100 |
| EMT | TIMP1 | 3.59E+08 | 9.27E+08 | 2.582119 | **EMT** | SRC | 0 | 5656378 | 100 |
| EMT | BCAR1 | 932791 | 2375990 | 2.547184 | **EMT** | STC1 | 0 | 14519117 | 100 |
| EMT | CAV1 | 1367988 | 3484054 | 2.546845 | **EMT** | STK10 | 0 | 4480711 | 100 |
| EMT | ICAM1 | 1629522 | 3912420 | 2.400962 | **EMT** | VEGFA | 0 | 3872900 | 100 |
| EMT | CD44 | 25276155 | 59551622 | 2.35604 | **EMT** | WNT7A | 0 | 670832.3 | 100 |
| EMT | LYPD3 | 6040985 | 13998921 | 2.317324 | **EMT** | AGR2 | 15675340 | 0 | 0 |
| EMT | PDGFB | 5245990 | 10736483 | 2.046608 | **EMT** | DAPK3 | 2601218 | 0 | 0 |
| EMT | S100P | 2799182 | 1300428 | 0.464574 | **EMT** | EFNA5 | 8629716 | 0 | 0 |
| EMT | PARVA | 2768496 | 1269462 | 0.458538 | **EMT** | GRHL2 | 1266536 | 0 | 0 |
| EMT | VPS4B | 2829590 | 1236636 | 0.437037 | **EMT** | MEGF8 | 339219 | 0 | 0 |
| EMT | PDCD6 | 11492703 | 4995651 | 0.43468 | **EMT** | TRIP6 | 3530861 | 0 | 0 |
| EMT | GIPC1 | 3260219 | 1336490 | 0.409939 | **EMT** | WASL | 226436 | 0 | 0 |
| EMT | APOA1 | 20915184 | 7098791 | 0.339408 |  |  |  |  |  |
| EMT | DDB1 | 10541455 | 3547273 | 0.336507 |  |  |  |  |  |
| EMT | HSPB1 | 1.39E+08 | 37965423 | 0.272424 |  |  |  |  |  |
| EMT | FBLN1 | 26139569 | 6328828 | 0.242117 |  |  |  |  |  |
| EMT | APOH | 4409311 | 811900.7 | 0.184133 |  |  |  |  |  |
| EMT | SLK | 6802197 | 368211.9 | 0.054131 |  |  |  |  |  |
